# A novel disulfidptosis-related lncRNAs signature for predicting survival and immune response in hepatocellular carcinoma

**DOI:** 10.18632/aging.205367

**Published:** 2024-01-04

**Authors:** Zhoubo Guo, Yan Xie, Li Zhang, Shuaichen Liu, Wentao Jiang

**Affiliations:** 1 The First Central Clinical School, Tianjin Medical University, Tianjin, China; 2 Department of Liver Transplantation, Tianjin First Central Hospital, Tianjin Medical University, Key Laboratory of Transplantation, Chinese Academy of Medical Sciences, Tianjin Key Laboratory for Organ Transplantation, Tianjin Key Laboratory of Molecular and Treatment of Liver Cancer, Tianjin, China

**Keywords:** disulfidptosis, lncRNAs, hepatocellular carcinoma, prognostic signature, immune

## Abstract

The accumulation of intracellular disulfides induces a novel and unique form of metabolic-related cell death known as disulfidptosis. A previous study revealed the prognostic value of a risk model of disulfidptosis-related genes in hepatocellular carcinoma (HCC). However, to date, no studies have investigated the relationship between disulfidptosis-related long non-coding RNAs (DRLs) and HCC. In this study, we collected and analyzed RNA sequencing data from 370 HCC samples to explore the DRLs in the tumorigenesis and development of HCC. By employing Lasso Cox regression and multivariate Cox regression analyses, we identified five prognostic DRLs, which were used to construct a prognostic signature. The signature was subsequently validated using receiver operating characteristic (ROC) curves, Kaplan-Meier analysis, Cox regression analyses, nomograms, and calibration curves. Gene Ontology (GO), Kyoto Encyclopedia of Genes and Genomes (KEGG), and gene set enrichment analysis (GSEA) were performed, revealing that the DRLs signature was associated with HCC and several cancer-related pathways. Furthermore, the DRLs signature showed correlations with the infiltration of M0 and M1 macrophages, immune-related functions, and multiple immune checkpoints, including PDCD1, LAG3, CTLA4, TIGIT, CD47, and others. Analysis using the tumor immune dysfunction and exclusion (TIDE) approach demonstrated that the DRLs signature could predict the response to immunotherapy. Finally, we screened potential chemotherapy drugs that could sensitize HCC. In conclusion, our novel DRLs signature provides valuable insights into predicting patient survival and immunotherapy responses.

## INTRODUCTION

According to the Global Cancer Statistics 2020, liver cancer accounted for nearly 906,000 diagnosed cases worldwide, with hepatocellular carcinoma (HCC) being the most prevalent subtype [1]. Hepatocellular carcinoma (HCC) arises from various factors, such as chronic infections like hepatitis B virus (HBV) or hepatitis C virus (HCV), alcohol misuse, non-alcoholic fatty liver disease, obesity, and diabetes, all playing significant roles in its development [2]. Although various treatment approaches for HCC exist, such as liver transplantation, surgical resection, ablation, radiation, and systemic therapy, the mortality rate of HCC remains high, with a 5-year survival rate of approximately 18% [3]. To improve therapeutic outcomes, it is crucial to explore novel biomarkers or predictive signatures that can predict patient survival and identify optimal candidates for different treatment approaches.

A previous study demonstrated that ectopic upregulation of disulfides induces high disulfide stress, leading to cell death, which can be counteracted by the reduced form of nicotinamide adenine dinucleotide phosphate (NADPH) [4, 5]. Recently, Liu et al. investigated the mechanism of disulfide-induced cell death by constructing a model combining ectopic high expression of solute carrier family 7 member 11 (SLC7A11) with glucose starvation. They observed that upregulation of SLC7A11 promotes disulfide accumulation, while glucose starvation depletes NADPH [6]. They further found that high disulfide stress induces the formation of disulfide bonds in actin cytoskeleton proteins, resulting in F-actin contraction and actin network collapse, leading to a novel form of cell death termed “disulfidptosis.” This type of cell death cannot be rescued by known cell death inhibitors [6]. Additionally, Zhong et al. discovered that treatment with thioredoxin reductase 1 (TXNRD1) inhibitors induces intracellular cystine accumulation and disulfidptosis of osteoclast precursors, thereby reducing bone loss. This process was promoted by nuclear factor of activated T-cells 1 (NFATc1)-mediated upregulation of SLC7A11 [7]. Moreover, Zhao et al. developed a disulfidptosis-related signature that showed promising predictive value for survival and immunotherapy outcomes in bladder cancer [8]. In addition, several studies revealed the potential relationships between disulfidptosis and cancers [9, 10]. Based on a previous study that identified 14 actin-related genes with increased disulfide bond formation following glucose starvation, including ACTB, ACTN4, CAPZB, CD2AP, DSTN, FLNA, FLNB, INF2, IQGAP1, MYH10, MYH9, MYL6, PDLIM1, and TLN1 [6]. These genes were defined as disulfidptosis-related genes (DRGs) and were included in our study.

Over 98% of the genome consists of noncoding sequences that produce numerous noncoding RNAs [11]. Long noncoding RNAs (lncRNAs), a subgroup of noncoding RNAs spanning at least 200 nucleotides, have been associated with the onset and advancement of diverse diseases [12]. Dysregulation of lncRNA expression has been reported in various malignancies, including HCC, due to epigenetic modification, transcriptional activation, and RNA processing, etc., [13]. LncRNAs primarily function by interacting with DNA, mRNA, microRNA, and proteins to engage in promoting or inhibiting tumorigenesis [13]. The expression of lncRNAs exhibits differences between HCC and normal liver tissues [14]. Research has confirmed the multifaceted involvement of lncRNAs in the onset and progression of HCC. Firstly, lncRNAs promote cell proliferation by upregulating cyclin D1 expression [15] and regulate apoptosis in HCC [16]. Secondly, lncRNAs regulate HCC cell invasion and metastasis by controlling epithelial-mesenchymal transition procession [17, 18] and interacting with miRNAs [19]. Thirdly, lncRNAs modulate the tumor microenvironment in HCC [20]. Fourthly, lncRNAs regulate liver cancer stem cells which are related to tumorigenesis and metastasis [21]. Several studies have identified ferroptosis- or cuproptosis-related lncRNAs as prognostic biomarkers for predicting patient survival in malignancies such as lung adenocarcinoma [22], glioma [23], colorectal cancer [24], breast cancer [25], and HCC [26, 27]. Until now, there have been no studies exploring the relationship between disulfidptosis-related lncRNAs (DRLs) and the prognosis of patients with HCC.

In this study, we employed RNA sequencing data sourced from The Cancer Genome Atlas-Liver hepatocellular carcinoma (TCGA-LIHC) database to identify DRLs. Following that, we actively developed and confirmed a distinctive signature reliant on DRLs to anticipate both the prognosis and the effectiveness of immunotherapy for individuals with HCC.

## RESULTS

### Recognition of the disulfidptosis-related prognostic lncRNA

**Figure 1:**
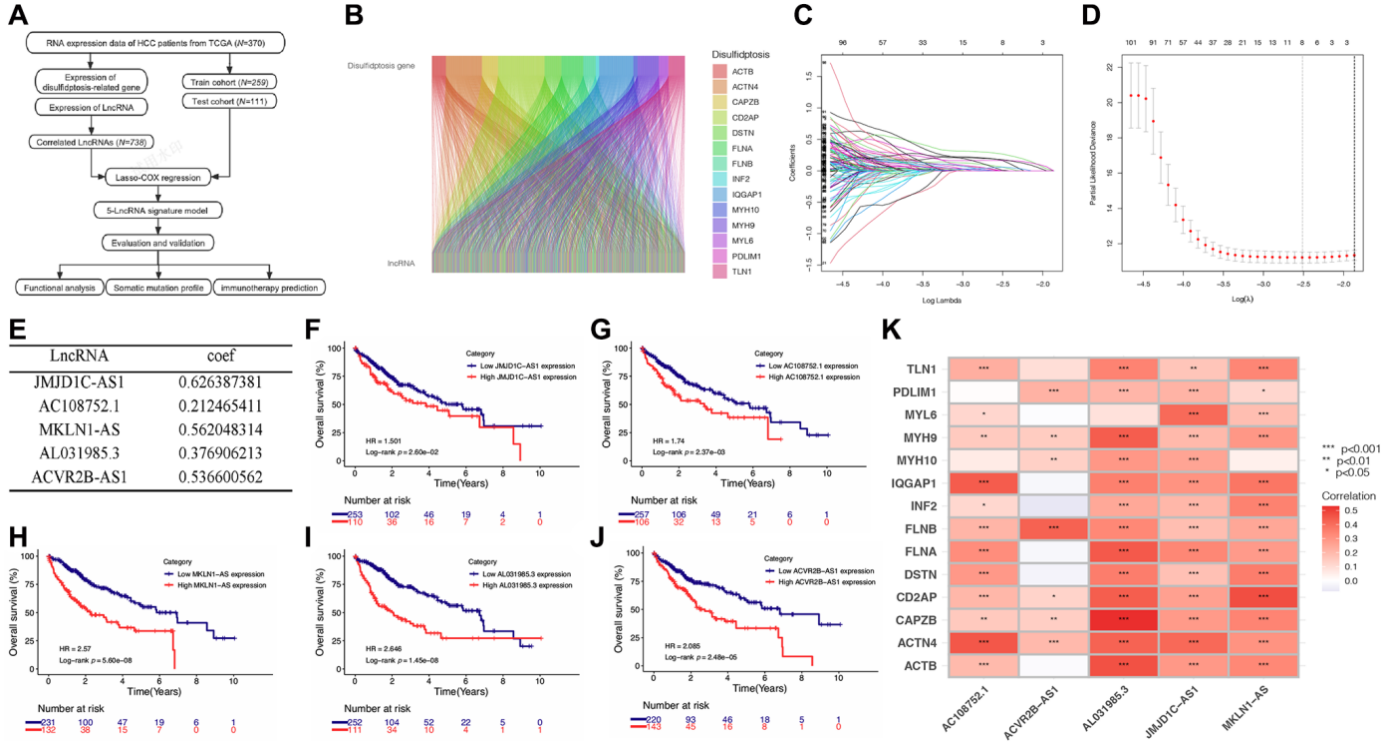
**Screening of prognostic DRLs in the TCGA-LIHC database.** (**A**) Data analysis flow of this study. (**B**) Correlation analysis between DRGs and lncRNAs. (**C**, **D**) Lasso Cox regression analysis. (**E**) Multivariate Cox regression analysis to determine the DRLs and their corresponding coefficients. (**F**–**J**) Kaplan-Meier (K-M) analyses of OS for five DRLs in the TCGA-LIHC cohort. (**K**) Correlations between DRGs and the five DRLs. Abbreviations: DRGs: disulfidptosis-related genes; DRLs: disulfidptosis-related lncRNAs. ^*^p < 0.05, ^**^p < 0.01, ^***^p < 0.001.

The process of data analysis is depicted in Figure 1A. We acquired the data encompassing both the expression profiles and clinical information from the TCGA-LIHC cohort and performed differential expression analysis and survival analysis of DRGs in this cohort (Supplementary Figures 1 and 2). Additionally, we investigated the protein expression levels of DRGs by referencing the Human Protein Atlas (HPA) (https://www.proteinatlas.org/) database and provided the immunohistochemistry findings for DRGs within HCC. (Supplementary Figure 3). Correlation analysis revealed that a total of 738 lncRNAs were associated with DRGs (Figure 1B). The results of the differential expression analysis of 738 disulfidptosis-related lncRNAs was showed in Supplementary Table 1. Subsequently, we partitioned the TCGA-LIHC cohort into a training set and a testing set, maintaining a ratio of 7:3. The baseline characteristics of the training and testing cohorts are presented in Table 1, demonstrating no significant differences between the two groups. We conducted univariate regression analysis in the training cohort and identified 218 lncRNAs significantly correlated with patient survival (Supplementary Table 2). After applying Lasso Cox regression for filtering (Figure 1C, 1D) and conducting multivariate Cox regression analysis, we identified the five best DRLs for constructing a prognostic signature: JMJD1C-AS1, AC108752.1, MKLN1-AS, AL031985.3, and ACVR2B-AS1 (Figure 1E). The survival analysis findings revealed a correlation between elevated expression levels of these five DRLs and a diminished overall survival (OS) among HCC patients (Figure 1F–1J). Furthermore, correlation analysis revealed a generally positive correlation between the five DRLs and DRGs in HCC (Figure 1K).

**Table 1:** Comparisons of patient characteristics between testing and training cohorts.

**Characteristics**	**Total (*n* = 370)**	**Testing cohort (*n* = 111)**	**Training cohort (*n* = 259)**	***p***
**Age**				
≤65	232 (62.7%)	65 (58.56%)	167 (64.48%)	0.336
>65	138 (37.3%)	46 (41.44%)	92 (35.52%)	
**Sex**				
Male	249 (67.3%)	83 (74.77%)	166 (64.09%)	0.059
Female	121 (32.7%)	28 (25.23%)	93 (35.91%)	
**Histologic grade**				
G1	55 (14.86%)	16 (14.41%)	39 (15.06%)	0.329
G2	177 (47.84%)	46 (41.44%)	131 (50.58%)	
G3	121 (32.7%)	42 (37.84%)	79 (30.5%)	
G4	12 (3.24%)	5 (4.5%)	7 (2.7%)	
Unknown	5 (1.35%)	3 (1.62%)	2 (1.08%)	
**T stage**				
T1	181 (48.92%)	55 (49.55%)	126 (48.65%)	0.953
T2	93 (25.14%)	27 (24.32%)	66 (25.48%)	
T3	80 (21.62%)	24 (21.62%)	56 (21.62%)	
T4	13 (3.51%)	3 (2.7%)	10 (3.86%)	
Unknown	3 (0.81%)	2 (1.8%)	1 (0.39%)	
**N stage**				
N0	252 (68.11%)	76 (68.47%)	176 (67.95%)	0.448
N1	4 (1.08%)	0 (0%)	4 (1.54%)	
Unknown	114 (30.81%)	35 (31.53%)	79 (30.5%)	
**M stage**				
M0	266 (71.89%)	78 (70.27%)	188 (72.59%)	>0.999
M1	4 (1.08%)	1 (0.9%)	3 (1.16%)	
Unknown	100 (27.03%)	32 (28.83%)	68 (26.25%)	
**TNM stage**				
I	171 (46.22%)	54 (48.65%)	117 (45.17%)	0.775
II	85 (22.97%)	25 (22.52%)	60 (23.17%)	
III	85 (22.97%)	22 (19.82%)	63 (24.32%)	
IV	5 (1.35%)	1 (0.9%)	4 (1.54%)	
Unknown	24 (6.49%)	9 (8.11%)	15 (5.79%)	

### Construction of the prognostic signature of DRLs in HCC

**Figure 2:**
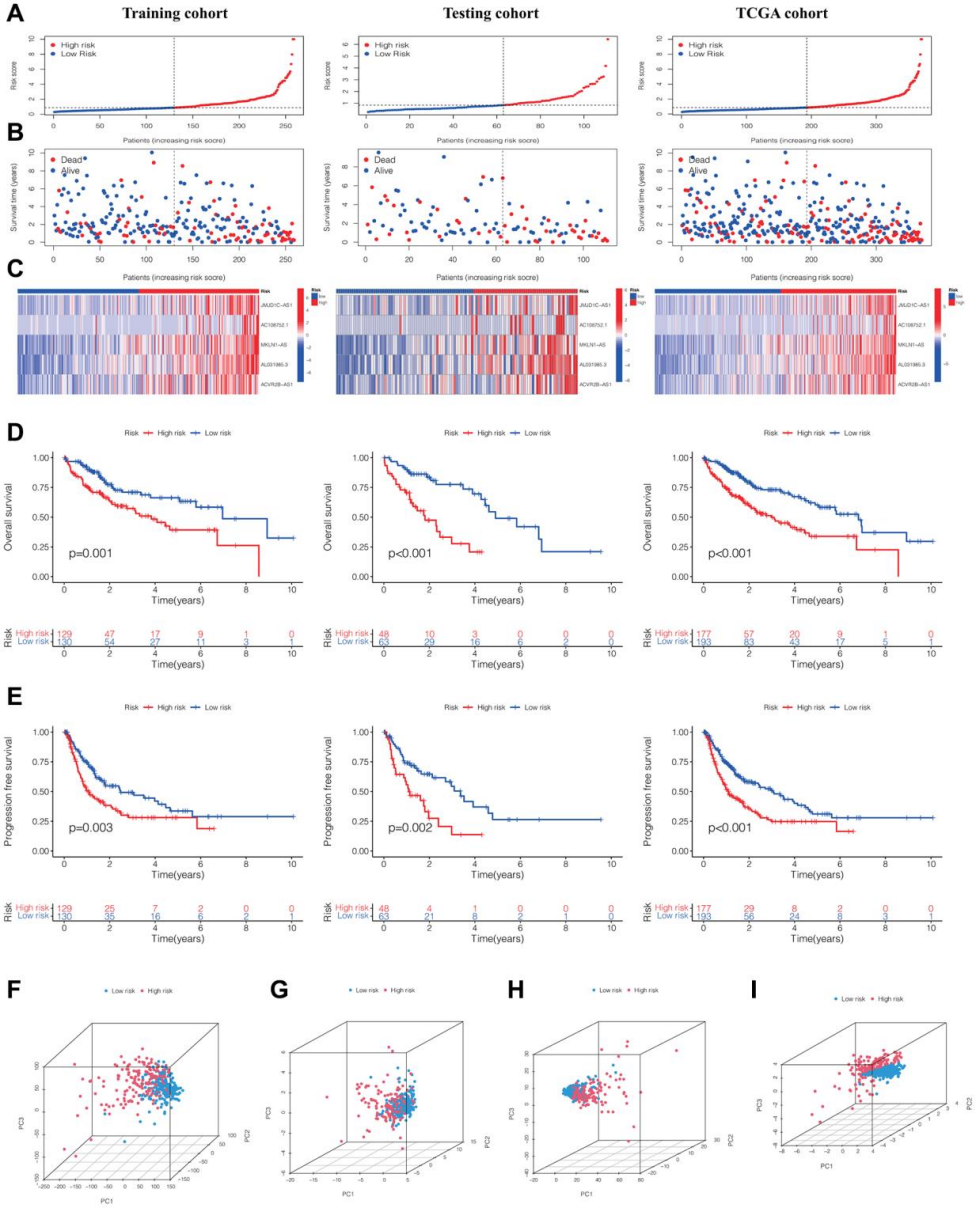
**Evaluation and validation of the DRLs signature in the training, testing, and TCGA cohorts.** (**A**) Distribution of normalized DRLs risk scores. (**B**) Survival status and survival time in relation to DRLs risk scores. (**C**) Heatmaps showing high- and low-risk groups. Kaplan-Meier analyses of OS (**D**) and PFS (**E**) for high- and low-risk groups. (**F**) PCA analysis of all genes. (**G**) PCA analysis of DRGs. (**H**) PCA analysis of all DRLs. (**I**) PCA analysis of DRLs risk score. Abbreviations: DRLs: disulfidptosis-related lncRNAs; OS: overall survival; PFS: progression-free survival; PCA: principal component analysis; DRGs: disulfidptosis-related genes.

Through the assessment of the expression levels of five DRLs in individuals with HCC, we computed a risk score for each patient. Subsequently, the training cohort, testing cohort, and TCGA cohort were stratified into low-risk and high-risk groups, with the division being determined by the median DRLs risk score calculated from the training cohort. (Figure 2A). Our analysis revealed that patients in the high-risk group exhibited poorer OS and higher mortality rates (Figure 2B), along with upregulated expression of the five DRLs (Figure 2C). Subsequently, Kaplan-Meier survival analysis was conducted and demonstrated that HCC patients in the high-risk group had shorter OS (Figure 2D) and progression-free survival (PFS) (Figure 2E) compared to patients in the low-risk group across all three cohorts.

Furthermore, upon contrasting the clustering of HCC patients according to the gene expression of all genes (Figure 2F), DRGs (Figure 2G), and all DRLs (Figure 2H), the principal component analysis (PCA) results demonstrated a clear distinction in the clustering of HCC patients into high- and low-risk groups (Figure 2I).

### The predictive performance of the prognostic signature of DRLs in HCC

**Figure 3:**
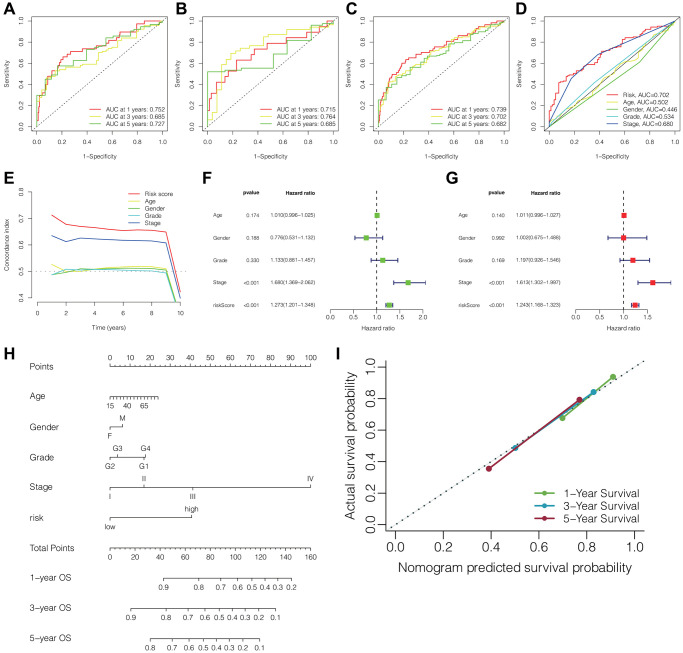
**Predictive performance of the DRLs signature and establishment of clinicopathologic nomogram.** ROC curves of the DRLs signature for predicting 1-, 3-, and 5-year OS in the training (**A**), testing (**B**), and TCGA cohorts (**C**). (**D**) ROC curves of the DRLs risk signature and clinical parameters for predicting 3-year OS in the TCGA cohort. (**E**) C-index of the DRLs signature and clinical parameters in the TCGA cohort. Univariate (**F**) and Multivariate (**G**) Cox regression analyses of the DRLs signature and clinical parameters. (**H**) Development of a prognostic nomogram for predicting patient survival in HCC. (**I**) Calibration curve of the prognostic nomogram. Abbreviations: DRLs: disulfidptosis-related lncRNAs; OS: overall survival; C-index: concordance index.

Figure 3A–3C illustrated the ROC curves of the DRLs signature for predicting the 1-, 3-, and 5-year OS in the training cohort, testing cohort, and TCGA cohort, respectively. The AUC values for the prediction of 1-, 3-, and 5-year OS were 0.752, 0.685, and 0.727 respectively in the training cohort, 0.715, 0.764, and 0.685 respectively in the testing cohort, and 0.739, 0.702, and 0.682 respectively in the TCGA cohort. Furthermore, the clinical ROC curve showcased the DRLs signature’s superior predictive accuracy for 3-year OS, displaying an AUC value of 0.702 within the TCGA cohort (Figure 3D). This finding was in line with the outcomes of the C-index curve analysis (Figure 3E). Moreover, the results of univariate and multivariate Cox regression analyses indicated the independence of the DRLs risk score as a prognostic indicator. (Figure 3F, 3G). Based on the results of the multivariate Cox regression analysis, we developed a prognostic nomogram for predicting patient survival in HCC (Figure 3H). The calibration curve in Figure 3I indicated that the nomogram performed well in accuracy for predicting 1-, 3-, and 5-year OS in HCC patients, and the C-index of the nomogram was 0.72 (95% CI 0.69–0.75).”

### Functional enrichment analysis of differential risk genes

**Figure 4:**
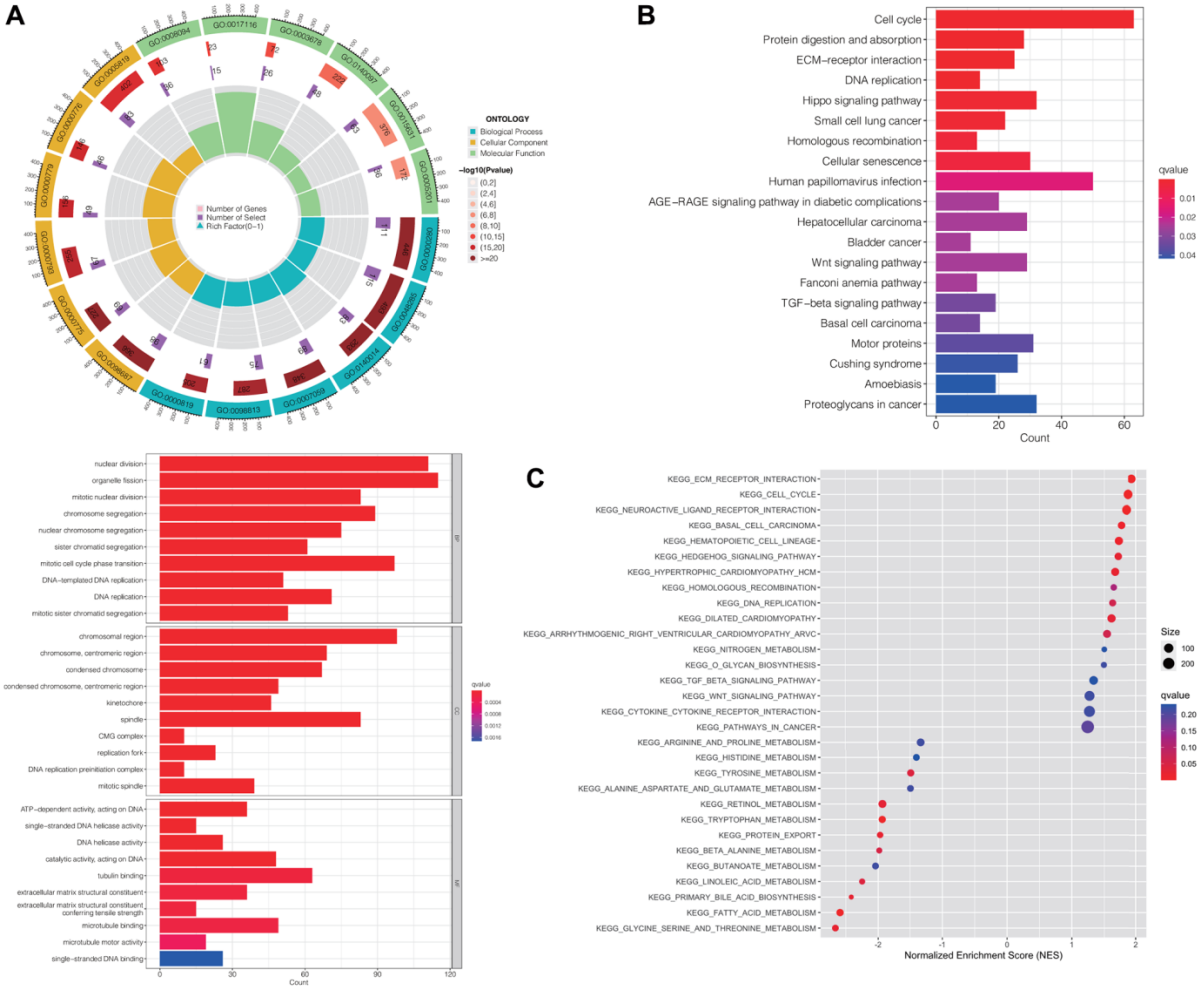
**Functional enrichment analyses of differentially expressed risk genes.** Results of GO (**A**), KEGG enrichment analysis (**B**), and GSEA (**C**). Abbreviations: GO: gene ontology; KEGG: Kyoto Encyclopedia of Genes and Genomes; GSEA: the gene set enrichment analysis.

We utilized the “limma” R package to extract the genes that were differentially expressed (DEGs) between the high-risk and low-risk groups. Subsequently, we conducted the Gene Ontology (GO) function analysis on these DEGs to explore their biological functions. Figure 4A illustrates that DEGs were enriched in biological processes (BP) such as “nuclear division”, “organelle fission” and “mitotic nuclear division”. In terms of cellular composition (CC), enrichment was observed in “chromosomal region”, “chromosome, centromeric region” and “condensed chromosome”. Additionally, for molecular function (MF), DEGs were associated with “ATP-dependent activity, acting on DNA”, “single-stranded DNA helicase activity” and “DNA helicase activity”. These observations indicated that the DEGs played a significant role in functions associated with chromosomes.

Furthermore, the Kyoto Encyclopedia of Genes and Genomes (KEGG) enrichment analysis revealed predominant enrichment of DEGs in pathways including “cell cycle”, “ECM-receptor interaction” and “protein digestion and absorption”. Notably, they were significantly correlated with pathways associated with hepatocellular carcinoma, cancer, wnt signaling, and TGF-beta signaling (Figure 4B).

For an in-depth investigation into the role of DRLs in the development and advancement of HCC, we conducted gene set enrichment analysis (GSEA). The results, consistent with the KEGG analysis, indicated that gene sets related to “ECM-receptor interaction,” “cell cycle,” and “neuroactive ligand receptor interaction” were enriched in the high-risk group. Additionally, cancer-related pathways, including “hepatocellular carcinoma,” “pathways in cancer,” “Wnt signaling pathway,” and “TGF-beta signaling pathway,” showed enrichment as well (Figure 4C).

### Mutation profiles of HCC and survival analysis

**Figure 5:**
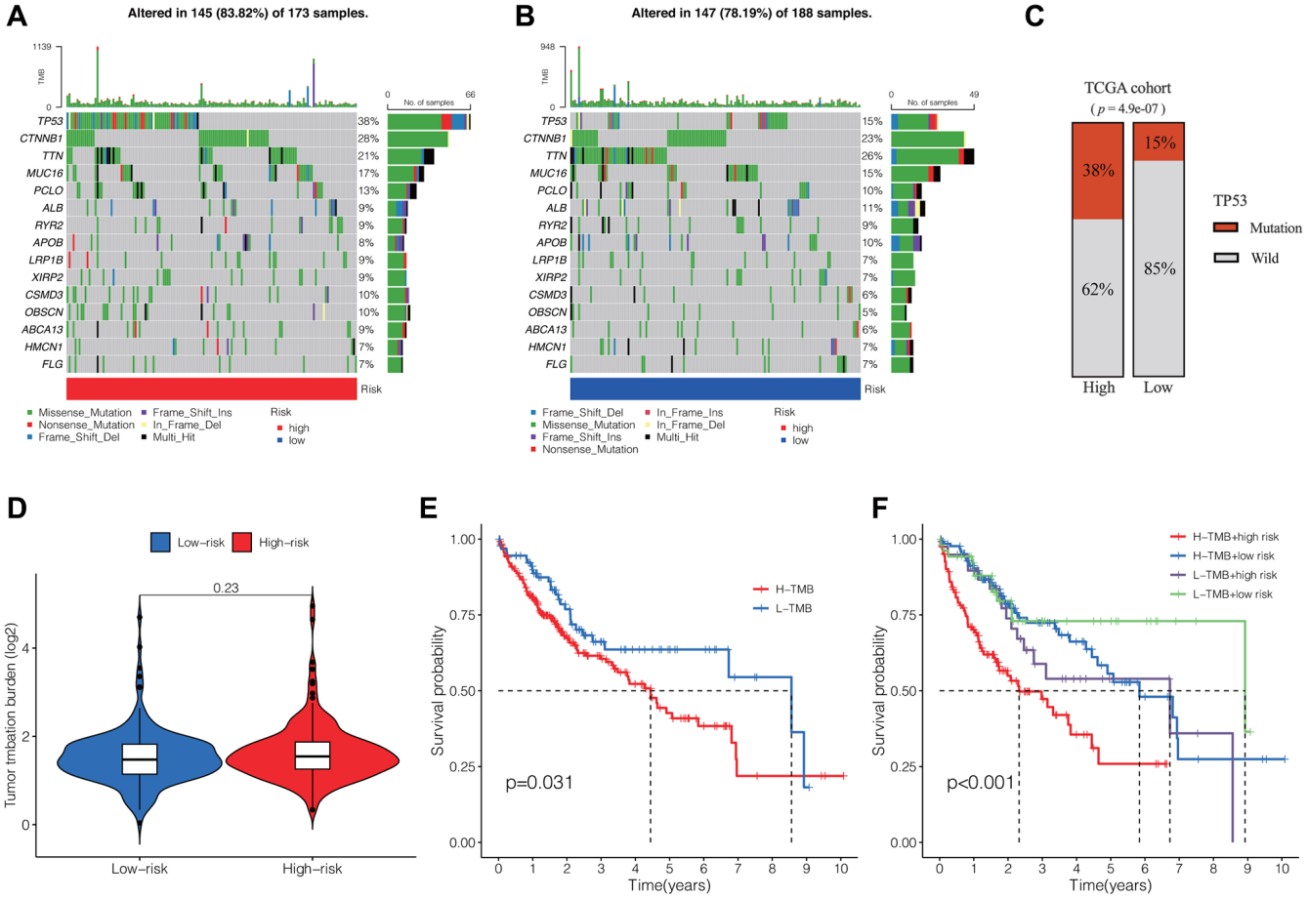
**Tumor somatic mutation profiles and survival analysis.** Waterfall plots showing the somatic mutation spectrum of HCC patients in the high-risk group (**A**) and low-risk group (**B**). (**C**) Comparison of TP53 mutation frequencies between the two groups. (**D**) Comparison of TMB between the two groups. (**E**) K-M analysis of TMB in HCC. (**F**) K-M analysis of TMB and DRLs risk scores. Abbreviation: TMB: tumor mutation burden.

We subsequently analyzed the somatic mutation spectrum of HCC patients in the high-risk and low-risk groups within the TCGA cohort. The results, displayed as waterfall plots in Figure 5A, 5B, revealed the top 15 genes with the highest mutation frequencies. TP53, CTNNB1, and TTN were identified as the three most frequently mutated genes. The high-risk group generally demonstrated elevated mutation frequencies across most genes, with TTN, however, showing an inverse trend. And patients in the high-risk group exhibited notably elevated TP53 mutation frequencies compared to those in the low-risk group (Figure 5C).

Additionally, we evaluated the tumor mutation burden (TMB) within the high-risk and low-risk groups, observing no substantial variances between the two groups (Figure 5D). However, the survival analysis revealed that patients with high TMB exhibited notably reduced overall OS compared to those with low TMB in cases of HCC. (Figure 5E). Moreover, when combining the DRLs risk score and TMB, we observed significant differences among four groups, with patients having high TMB and risk score exhibiting the shortest OS (Figure 5F).

### Correlations between DRLs signature and tumor immune microenvironment and immunotherapy outcomes

**Figure 6:**
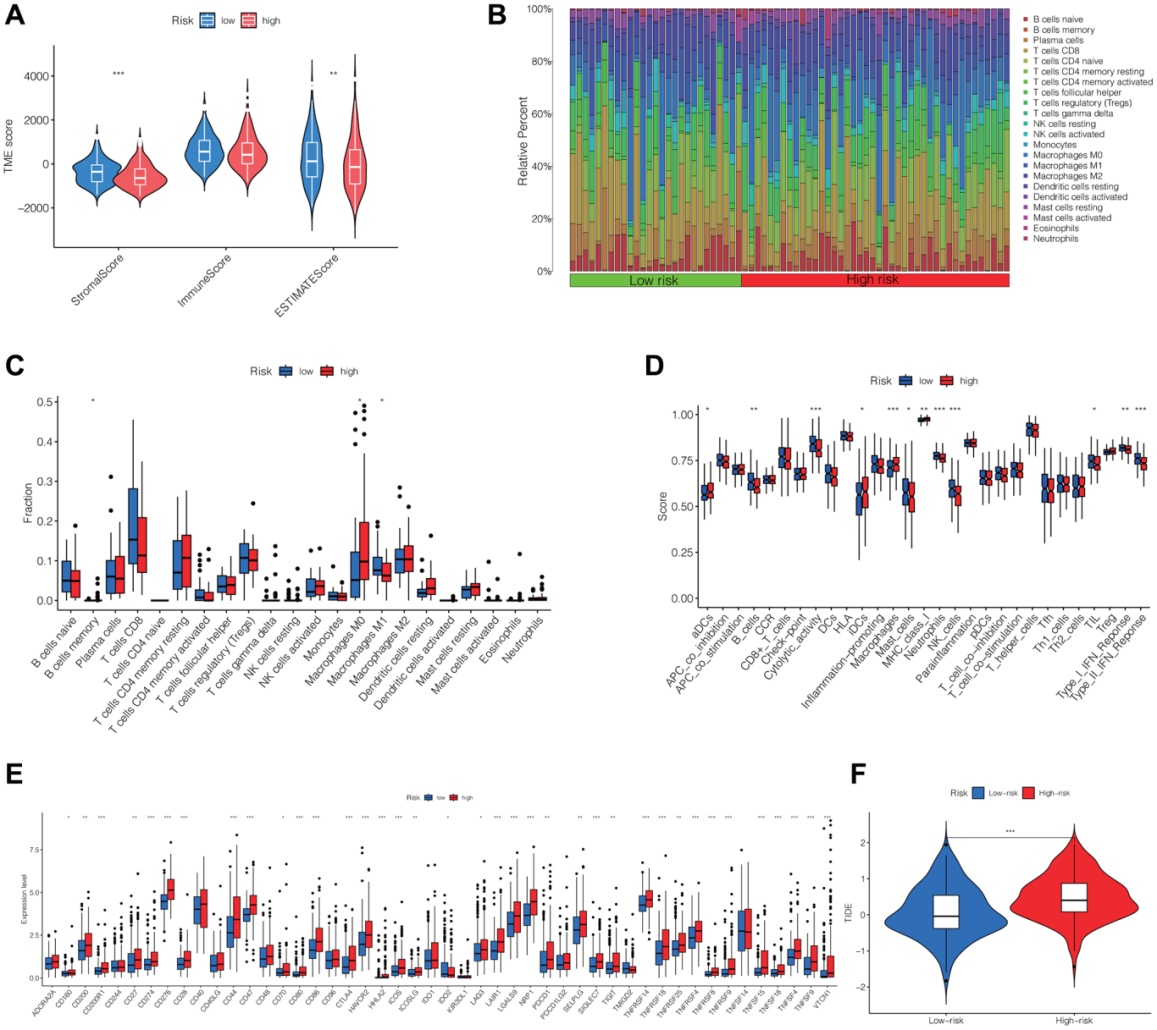
**Differential analysis of immune cells, immune function, immune checkpoints and immunotherapy outcome between high- and low-risk groups.** (**A**) The stromal, immune, and ESTIMATE scores between the two groups. (**B**, **C**) Differences in the infiltration of immune cells between the two groups. (**D**) Comparison of immune functions between two groups. (**E**) Differences in the expression of immune checkpoints between the two groups. (**F**) TIDE in the two groups. Abbreviation: TIDE: evaluation of tumor immune dysfunction and exclusion. ^*^p < 0.05, ^**^p < 0.01, ^***^p < 0.001.

Using the “estimate” R package, we computed the stromal, immune, and ESTIMATE scores for every individual in the TCGA cohort. The Wilcoxon test findings revealed markedly reduced stromal and ESTIMATE scores among patients in the high-risk group compared to those in the low-risk group, whereas no significant difference was observed in the immune score between the two groups (Figure 6A). Furthermore, we assessed the immune cell composition within HCC patients in the TCGA cohort and contrasted the immune cell scores between the two risk groups. These results were presented as histograms and box plots. We observed significant differences in the content of B cell memory, macrophage M0, and macrophage M1 between the two risk groups (Figure 6B, 6C). Figure 6D demonstrated significant differences in immune pathways between the high-risk and low-risk groups, including aDCs, B_cells, Cytolytic_activity, iDCs, Macrophages, Mast_cells, MHC_class_I, Neutrophils, NK_cells, TIL, Type_I_IFN_Response, and Type_II_IFN_Response. Additionally, we examined the variations in expression of immune checkpoints between the two risk groups and discovered statistically significant differences in the majority of the results, encompassing the expression of PDCD1, LAG3, CTLA4, TIGIT, CD47, and various others. (Figure 6E). Finally, we evaluated the TIDE (Tumor Immune Dysfunction and Exclusion) in both the high-risk and low-risk groups, revealing that the high-risk group exhibited notably elevated TIDE scores compared to the low-risk group. This result implied that individuals within the high-risk group might possess an increased likelihood of immune evasion and experience comparatively reduced advantages from immunotherapy (Figure 6F).

### Correlation between DRLs signature and drugs for HCC

**Figure 7:**
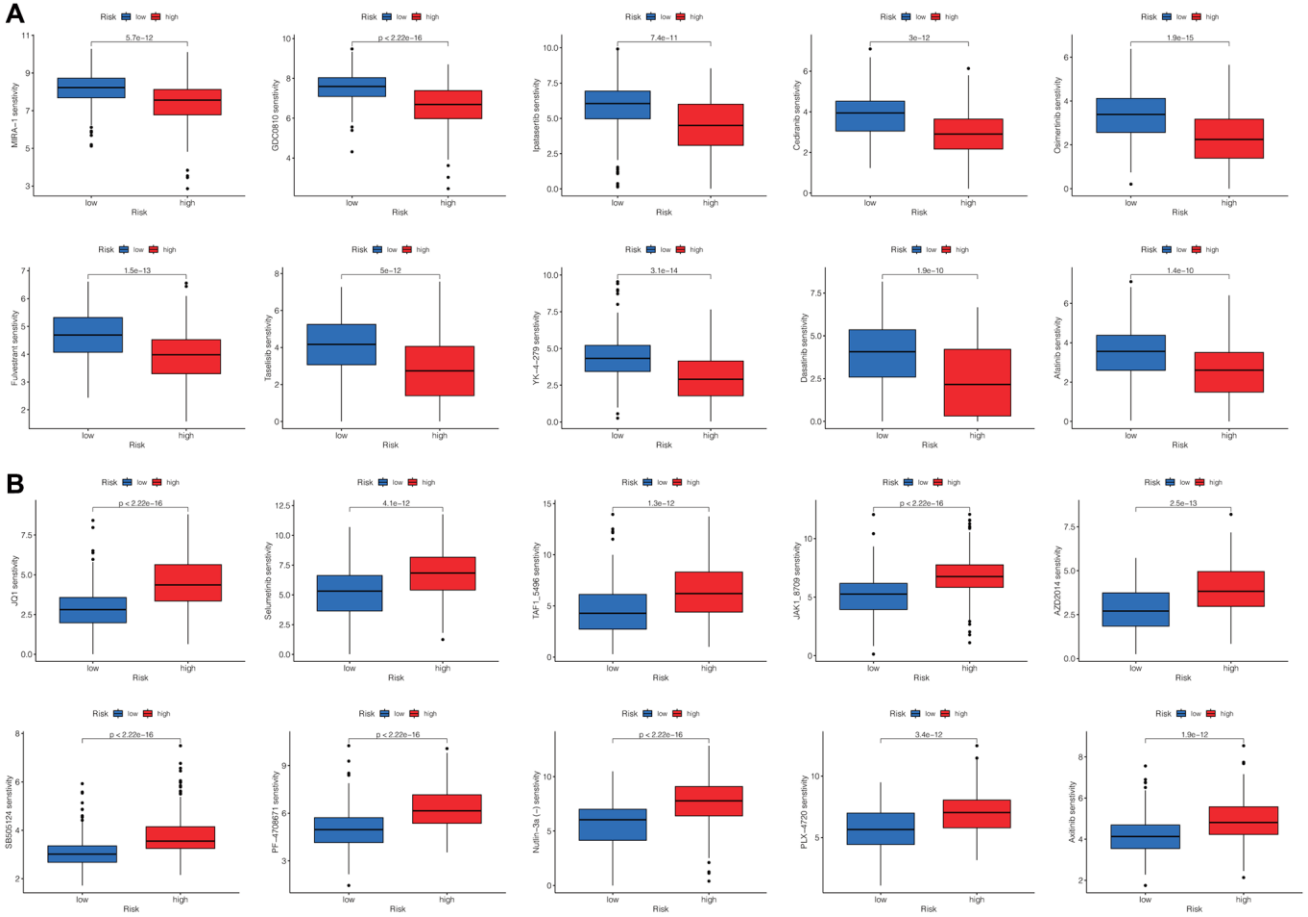
**Screening the potential sensitive chemotherapy drugs for HCC.** The top 10 most significantly sensitive drugs for the high-risk group (**A**) and the low-risk group (**B**), respectively.

Additionally, we compared drug sensitivity scores between the two risk groups. A lower score indicates higher drug sensitivity. We totally observed discrepant sensitivities to 102 drugs between the two risk groups. We presented the top 10 most significantly differentially sensitive drugs for the high-risk group (Figure 7A) and the low-risk group (Figure 7B), respectively.

## DISCUSSION

In recent years, multidisciplinary treatments have improved the prognosis of patients with HCC, but the survival rate remains poor. A previous study revealed that HCC patients benefited from the administration of immune checkpoint inhibitors (ICIs), such as anti-PD-1, anti-PD-L1, and anti-CTLA-4, leading to FDA approval of immunotherapy as a first-line or second-line treatment [28]. However, the lack of an appropriate patient screening method has resulted in the inefficient therapeutic effect of immunotherapy for HCC. In this study, we identified five DRLs and constructed a prognostic signature to predict patient survival and the outcome of immunotherapy. This signature can be used to screen optimal HCC patients for ICI treatment.

Disulfidptosis is a novel mode of metabolic-related cell death induced by the ectopic accumulation of intracellular disulfides [6]. The lack of cystine is crucial for inducing ferroptosis in cancer cells, and many cancers upregulate SLC7A11 to obtain a sufficient supply of cystine and avoid ferroptosis [29]. However, Liu et al. found that overexpression of SLC7A11 resulted in the accumulation of cystine, leading to a striking loss of NADPH when combined with glucose starvation [6]. The depletion of intracellular NADPH, caused by the accumulation of cystine, triggered the massive formation of disulfide bonds in actin cytoskeleton protein molecules, resulting in the contraction of F-actin and ultimately leading to disulfidptosis [6]. The authors determined 14 actin cytoskeleton proteins which upregulated at least 1.5-fold of disulfide bonds after the treatment of glucose starvation of SLC7A11 overexpressed cells, including ACTB, ACTN4, CAPZB, CD2AP, DSTN, FLNA, FLNB, INF2, IQGAP1, MYH10, MYH9, MYL6, PDLIM1, and TLN1 [6]. We identified five prognostic lncRNAs which correlated with these 14 genes and explored the potential mechanism and prognostic value of the DRLs-related model in HCC.

Considering that lncRNAs play a vital role in the occurrence and progression of cancers and its prognostic value has been explored in many cancer types [22–27]. We utilized RNA sequencing data from the TCGA database to study the correlation between DRLs and HCC patient survival, tumor immune microenvironment (TME), and immunotherapy efficacy. We identified five prognostic DRLs, including JMJD1C-AS1, AC108752.1, MKLN1-AS, AL031985.3, and ACVR2B-AS1, and constructed a prognostic risk-scoring signature.

This DRLs risk signature effectively stratified HCC patients into high- and low-risk groups. The risk score emerged as an independent prognostic indicator for HCC, with patients in the high-risk group exhibiting significantly worse OS and PFS compared to those in the low-risk group. Furthermore, we provided a DRLs-related clinicopathological nomogram to precisely and straightforwardly predict 1-, 3-, and 5-year survival of HCC patients.

Enrichment analysis revealed a positive correlation between the DRLs risk signature and multiple pathways, including “cell cycle”, “ECM-receptor interaction”, “hepatocellular carcinoma”, “Wnt signaling pathway”, and “TGF-beta signaling pathway”. Dysregulation of the cell cycle and ECM-receptor interaction pathways promotes the proliferation and migration of cancer cells. Meanwhile, it has been reported that the Wnt and TGF-beta signaling pathways are associated with the development and progression of HCC [30, 31]. The overactivation of these pathways was a contributing factor to the unfavorable prognosis observed in the high-risk group of patients.

TME is closely related to the occurrence and development of malignancies. We further investigated the correlations between the DRLs risk score and infiltrating immune cells, immune-related functions, immune checkpoints, immune escape, and the outcome of immunotherapy in HCC. Specifically, our findings revealed a significant correlation between the risk score and the content of B cell memory, macrophage M0, and macrophage M1. M0 macrophages are resting state macrophages that polarize into M1 macrophages upon stimulation by interferon and/or lipopolysaccharide. M1 macrophages play a crucial role in antitumor immune responses by presenting antigens to adaptive immune cells, releasing proinflammatory cytokines, and phagocytosing tumor cells [32, 33]. Despite being in a resting state, M0 macrophages have been found to function in a tumorigenic role, and the content of M0 macrophages negatively correlates with the prognosis of glioma [34] and bladder cancer [35]. Consistent with previous findings, our results indicated that a high-risk score correlated with a high content of M0 macrophages and a low content of M1 macrophages, which might contribute to the poor survival of patients in the high-risk group. Additionally, our findings unveiled that an elevated risk score was associated with diminished antitumor immune-related functions, increased expression of diverse immune checkpoints, and a high TIDE score. These findings suggested that DRLs played a role in regulating the TME and antitumor immune response, thereby influencing tumorigenesis and progression. Furthermore, we also screened some chemotherapy drugs by analyzing drug sensitivity in the two groups.

There are some limitations in this study. Firstly, the DRLs risk model was constructed and validated using the TCGA database, and it lacked external validation to verify feasibility. Additionally, the results of this study needed to be verified by experimental tests in further studies.

In conclusion, we have developed a prognostic signature for DRLs to predict patient survival and select optimal candidates for immunotherapy in HCC. Furthermore, this study provides novel insights into the potential mechanisms of DRLs in regulating the formation of the TME in HCC.

## MATERIALS AND METHODS

### Data acquisition and processing

The RNA sequencing data and relevant clinical details of 370 patients diagnosed with HCC were obtained from the TCGA-LIHC database, accessible at https://portal.gdc.cancer.gov/. The clinical information included age, sex, histologic grade, tumor node, metastasis (TNM) stage, and survival data. As a control, RNA sequencing data from 50 normal samples from the same database were extracted for comparative analysis. The average value was used to integrate the sequencing data of different tumor samples from the same patient. We obtained a total of 19,938 mRNAs and 16,877 lncRNAs after classification of the transcriptomic data for further analysis. To establish and validate the prognostic signature, we employed the “caret” R package to randomly partition patients from the TCGA-LIHC database into two groups. This division resulted in 259 patients designated for the training cohort and 111 patients allocated to the testing cohort, maintaining a ratio of 7 to 3. Furthermore, we collected the simple nucleotide variation data from the TCGC-LIHC database to calculate the TMB and analyze gene mutation content in HCC patients.

### Construction and validation of the disulfidptosis-related lncRNA prognostic signature in HCC

As mentioned earlier, the DRGs included ACTB, ACTN4, CAPZB, CD2AP, DSTN, FLNA, FLNB, INF2, IQGAP1, MYH10, MYH9, MYL6, PDLIM1, and TLN1. We conducted correlation analysis to select 738 lncRNAs meeting the criteria of a correlation coefficient exceeding 0.4 and a significance level of *p* < 0.001. The results were visualized using the “ggalluvial” and “ggplot2” R packages [36]. Among these lncRNAs, univariate Cox analysis was performed to identify 218 prognostic lncRNAs in the training cohort. Finally, Lasso Cox regression analysis was conducted using the “glmnet” R package [37], and five lncRNAs were determined as prognostic DRLs. The risk score attributed to each patient was computed via the subsequent formula, incorporating the coefficient and expression value of each of these five lncRNAs: DRLs risk score = expression of JMJD1C-AS1 × 0.626387381 + expression of AC108752.1 × 0.212465411 + expression of MKLN1-AS × 0.562048314 + expression of AL031985.3 × 0.376906213 + expression of ACVR2B-AS1 × 0.536600562.

Using the median risk score obtained from the training cohort, patients in the training, testing, and TCGA cohorts were categorized into high-risk and low-risk groups. To assess the performance of the model, we conducted receiver operating characteristic (ROC) curve analysis, time-dependent ROC curve analysis, Kaplan-Meier (K-M) analysis, and principal component analysis (PCA) using the “timeROC,” “survival,” and “survminer” R packages respectively.

### Construction and validation of a clinicopathological nomogram

Univariate and multivariate Cox regression analyses were performed to assess the potential of the DRLs risk score as an independent prognostic indicator for the survival of HCC patients. Subsequently, the “rms” R package was utilized to generate a nomogram associating the DRLs risk score [38], and a corresponding calibration curve was plotted to assess the accuracy of the nomogram in predicting the survival of HCC patients.

### Functional enrichment analysis

First, we identified the differentially expressed genes (DEGs) distinguishing the high- and low-risk groups within the TCGA cohort using the “limma” R package. The “clusterProfiler” R package was utilized to perform the Gene Ontology (GO) and Kyoto Encyclopedia of Genes and Genomes (KEGG) analysis [39]. And to identify the differences of biological function between high- and low-risk groups, the gene set enrichment analysis (GSEA) was implemented based on the gene set of “c2.cp.kegg.v2023.1.Hs.symbols.gmt” via the “clusterProfiler” R package [39].

### Tumor somatic mutation analysis

We utilized waterfall plots generated by the “maftools” R package to visualize the differences in gene mutation frequencies between the high-risk and low-risk groups [40]. Furthermore, we employed the “limma” R package to assess the disparity in TMB between the two groups. Additionally, we performed Kaplan-Meier analysis to determine whether TMB or TMB combined with the risk score correlated with the survival of HCC patients using the “survival” and “survminer” R packages.

### Assessment of immune cell infiltration, immune microenvironment, and immunotherapy outcome

Using the “estimate” R package, we assessed the infiltration of immune cells and obtained the TME score for each HCC sample. The Wilcoxon test was performed to compare the TME scores and the expression of immune checkpoints between the high-risk and low-risk groups. We utilized the “CIBERSORT” algorithm to evaluate the differences in immune cell infiltration between the two groups [41]. Furthermore, we employed the tumor immune dysfunction and exclusion (TIDE) algorithm to calculate the TIDE score [42], which indicated the outcome of immunotherapy, and compared the difference in TIDE score between the high-risk and low-risk groups.

### Prediction of drug sensitivity for HCC

We used the “oncopredict” R package [43] to compute the scores of drug responses in the TCGA cohort for 198 drugs. The differences in drug sensitivities between the high-risk and low-risk groups were compared using the Wilcoxon test.

### Statistical analysis

We utilized R software (version 4.2.2, http://www.R-project.org) for all data analyses. Survival analyses were performed using the Kaplan-Meier curve, log-rank test, and univariate and multivariate Cox regression analyses. Differences between two groups were analyzed using Student’s *t*-test or the Wilcoxon test. The Chi-square test was employed to compare the baseline characteristics between the training and testing groups. Furthermore, correlation analyses were conducted using Pearson correlation analysis. A two-tailed *p*-value < 0.05 was considered statistically significant. ^*^p < 0.05, ^**^p < 0.01, ^***^p < 0.001.

## Supplementary Materials

Supplementary Figures

Supplementary Table 1

Supplementary Table 2
