# Supplementary material for: A novel disulfidptosis-related lncRNAs signature for predicting survival and immune response in hepatocellular carcinoma
Source: Aging (Albany NY). 2024 Jan 4;16(1):267–84. doi: 10.18632/aging.205367 (PMC10817373; doi:10.18632/aging.205367)
Supplement: Supplementary Figures [file aging-16-205367-s001.pdf]

SUPPLEMENTARY FIGURES

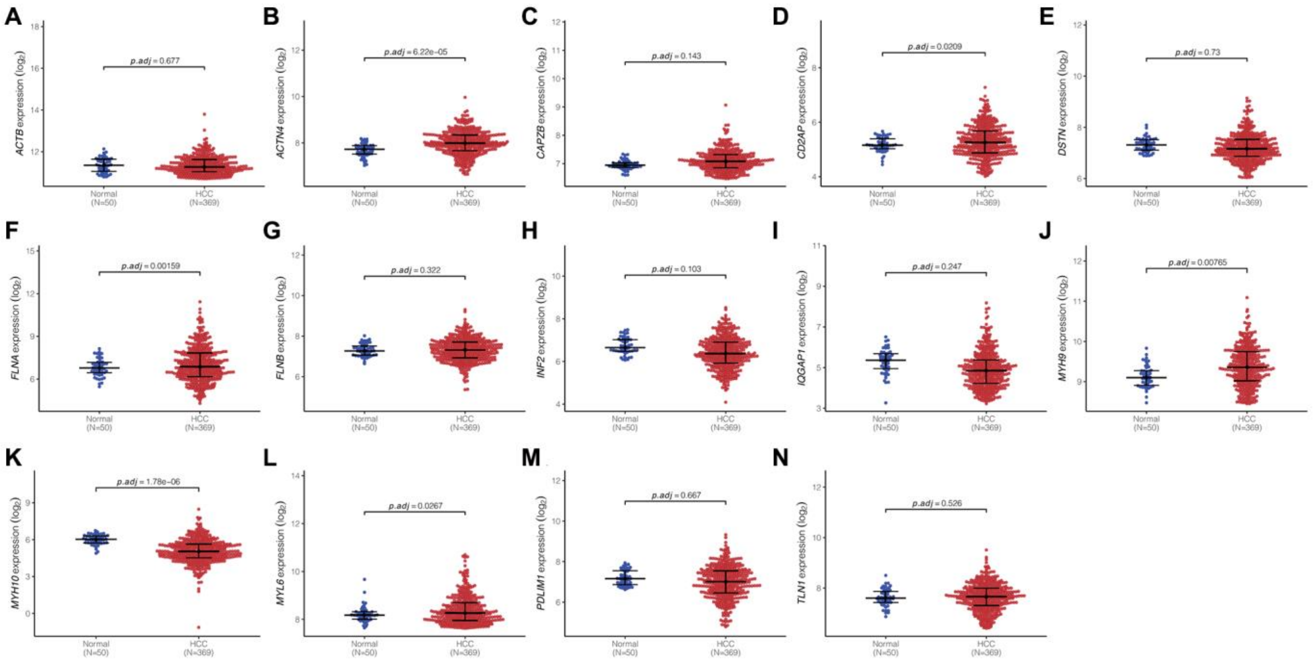

Supplementary Figure 1. Differential analyses of 14 DRGs in HCC, including ACTB (**A**), ACTN4 (**B**), CAPZB (**C**), CD2AP (**D**), DSTN (**E**), FLNA (**F**), FLNB (**G**), INF2 (**H**), IQGAP1 (**I**), MYH9 (**J**), MYH10 (**K**), MYL6 (**L**), PDLIM1 (**M**), TLN1 (**N**).

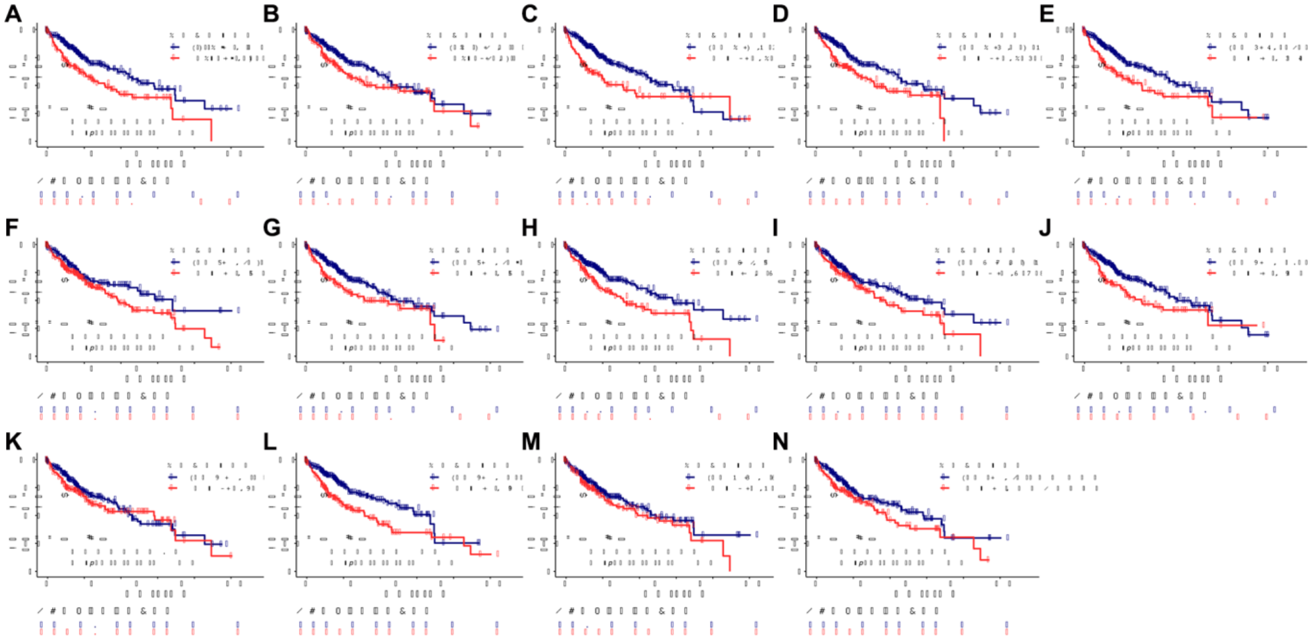

Supplementary Figure 2. Survival analyses of 14DRGs in HCC, including ACTB (**A**), ACTN4 (**B**), CAPZB (**C**), CD2AP (**D**), DSTN (**E**), FLNA (**F**), FLNB (**G**), INF2 (**H**), IQGAP1 (**I**), MYH9 (**J**), MYH10 (**K**), MYL6 (**L**), PDLIM1 (**M**), TLN1 (**N**).

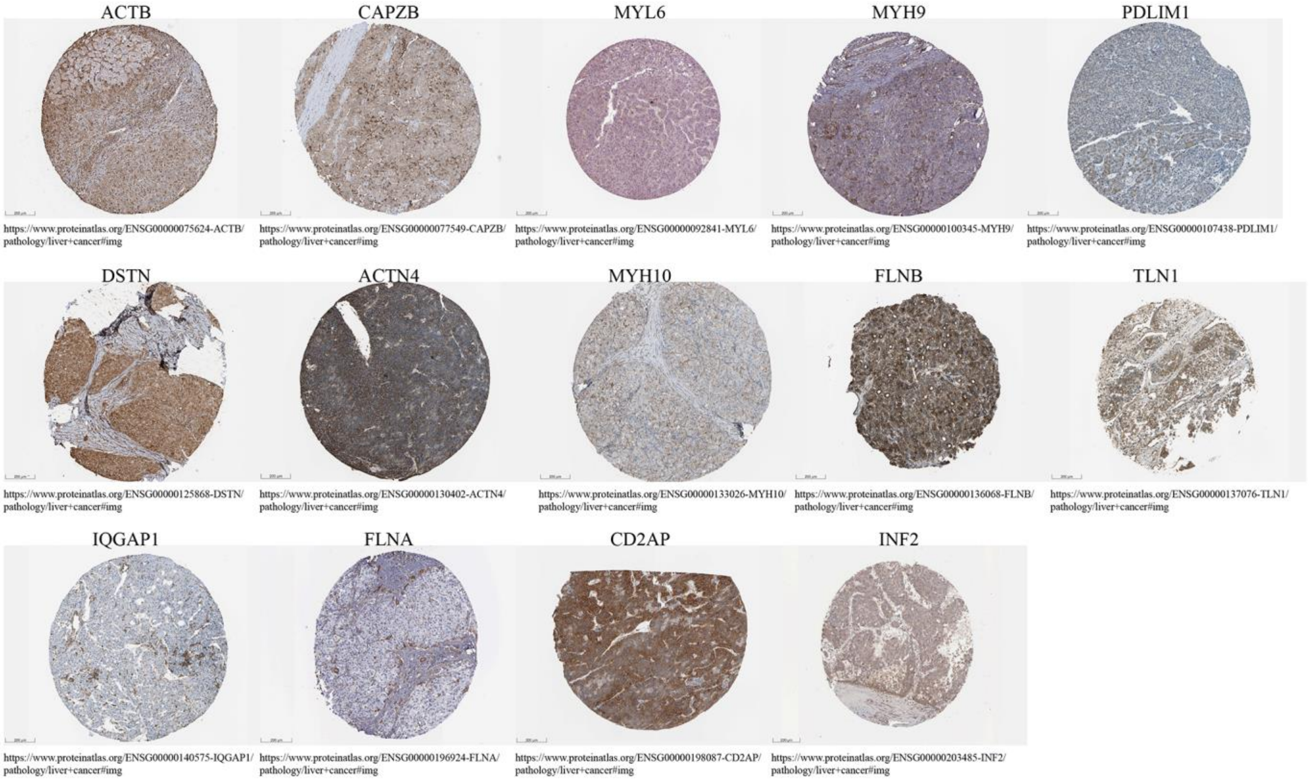

Supplementary Figure 3. The immunohistochemistry pictures of 14 DRGs in HCC from the Human Protein Atlas (HPA) database.
